# Anthropometric indices, body function, and physical fitness reference values for Tibetan ethnic children aged 6–17 residing at 3,650 meters above sea level

**DOI:** 10.3389/fnut.2022.1036470

**Published:** 2022-10-14

**Authors:** Xiaowei Ma, Yong Mao, Jian Wang, Zewangzhandui, Xiaomei Wang

**Affiliations:** ^1^College of Health and Exercise Science, Tianjin University of Sport, Tianjin, China; ^2^Education (Sports) Bureau of Jomda County, Chamdo, China

**Keywords:** growth, BMI, blood pressure, forced vital capacity, GAMLSS, LMS

## Abstract

**Objectives:**

It is known that high altitude influences the growth metrics of high-altitude residents. Using a WHO-recommended standard, the research aimed to establish growth and development reference values for children of Tibetan ethnicity between the ages of 6 and 17 years old.

**Methods:**

The measurements took place in Jomda County, Tibet with an average altitude of 3,650 m above sea level. A total of 3,955 observations (1,932 boys and 2,023 girls) were utilized to model the centile estimations. Included in the measurements are height, weight, body mass index, heart rate, blood pressure, forced lung capacity, sit and reach, and standing long jump. The measurements were modeled using the generalized additive models for location, scale, and shape (GAMLSS). Models were fitted with suitable distributions and locally smoothed using the P-spline for each GAMLSS hyper-parameter. Using the smallest Schwarz Bayesian criterion, the optimal model for each measurement was selected. After model adjustment, centile estimations were calculated for each model.

**Results:**

Compared to the height reference values at the 50th percentile for multi-ethnic Chinese children residing at low altitudes, Tibetan ethnic children exhibit apparent stunted growth. In terms of forced vital capacity, it is remarkable that Tibetan ethnic children lag behind multi-ethnic Chinese children residing at low altitudes. Heart rate and blood pressure regulation are generally normal. Centile estimations are provided in this article and tabulated centiles (1p, 3p, 5p, 15p, 25p, 50p, 75p, 85p, 95p, 97p, 99p) in Chinese, Tibetic, and English are openly available in FigShare (doi: 10.6084/m9.figshare.20898196.v1).

**Conclusion:**

This study established the first GAMLSS based growth and development reference values for Tibetan ethnic children aged 6–17. These reference values have numerous clinical and scientific applications. We offer Chinese policymakers with practical initiatives to further enhance the health of Tibetan ethnic children.

## Introduction

Hypoxia is a drop in the normal level of tissue oxygen tension, and it is well-known that children permanently exposed to hypoxia at altitudes greater than 3,000 m above sea level (hereinafter referred to as “m”) exhibit phenotypical adaptation and different growth patterns (1). Existing global and local data suggest that at 2,000 m, the adverse effect of altitude on child height growth would intensify (2), and Tibetan children residing above 3,500 m had a two-to-sixfold increased risk of stunting compared to those residing at 3,000 m (3). The adaptation of blood pressure regulation at high altitude is another consequence of hypoxia with major epidemiological significance. According to a recent meta-analysis, the prevalence of hypertension among permanent high-altitude residents is 33.0% (4), and adults of Tibetan ancestry are more susceptible to developing hypertension (5). Accumulated evidence demonstrates unequivocally that the generic growth of high-altitude residents in Tibet are distinct, and it is of considerable epidemiological significance to track the growth of Tibetan ethnic children exposed to high altitude.

Clinical evaluations of a child’s health are based on growth measurements. Typically, growth reference values are set for the pediatric population in order to evaluate their generic health state, nutritional deficiency, and developmental pattern. The implementation of health policy in Tibet has historically been less optimal due to cultural, environmental, geographic, and economic issues, and as a result, the physical development of Tibetan ethnic children (6) and adolescents (7, 8) is below that of Han ethnic children and adolescents. The fact that delayed growth in young Tibetan children, as measured by height, is independent of socioeconomic factors and has been suggested to be attributable to the altitude effect (3) highlights the need to monitor different growth patterns among Tibetan children. Clearly, reference values derived from Han ethnic populations residing at sea level may not apply to Tibetan ethnic minority.

There are few published anthropometric studies of Tibetan children (9). Aside from a single systematic study on Tibetan pre-school children (6), we are unaware of any growth reference values or physical fitness criterion for Tibetan school-aged children, underscoring the need for a standard based on scientific measurement and statistical analysis. We argue that the statistical method involved in developing a valid growth-for-age reference values is one of the primary reasons for this apparent research gap that has not been addressed. Cole commented that the creation of growth curves has always been somewhat of a black art (10). Historically, growth curves were developed using z-scores, but the underlying assumption for z-scores is sometimes violated for growth-related data whose distribution may be skewed and kurtotic. For example, Mary et al. (11) reported lung function reference values for Hong Kong children and adolescents. Their data, on the other hand, are manifestly non-Gaussian, hence their z-scores and reference values cannot be interpreted correctly. A few of statistical pioneers have subsequently tackled this problem (12, 13), and the solutions have been refined and integrated into an advanced statistical method known as the generalized additive model for location, scale, and shape (GAMLSS) (14). Briefly, GAMLSS enables fitting non-Gaussian growth-related variable to a particular distribution, and all percentile centile curves can be modeled without crossing in a continuous age.

To further improve the health of Tibetan ethic children, the Chinese government legislated a series of targeted special fiscal aid, and in 2019 we were tasked with examining the growth and development pattern of Tibetan ethnic children in Jomda County (15). In this study, Tibetan school children residing at an average altitude of 3,650 m were measured and evaluated for their anthropometric indices, body function, and physical fitness in an effort to provide accurate data to support the development of growth and development standards for Tibetan children residing in high-altitude areas. Reference values have critical epidemiological, physiological, and training implications for promoting the healthy growth of Tibetan children. Ultimately, this study aimed to employ GAMLSS to establish centile estimations that are easily interpretable and readily usable by both academics and practitioners.

## Materials and methods

### Participants

From August through October of 2019, Tibetan children from 15 schools in Jomda County, at an average altitude of 3,650 m were selected for testing based on the premise of random total population sampling. This cross-sectional study was approved by the Tianjin University of Sport and the legal guardian of each children provided consent before data collection.

According to the seventh national population census (16), Tibetan ethnic minority accounts for more than 99.9% of the total population in Jomda County; therefore, it is presumed that the sample population in this study consisted of Tibetan ethnic children. A total of 4,765 children were measured. Jomda County is situated in a rural region of Tibet, and children are enrolled in schools due to local conditions. As a result, there are a few individuals under the age of 6 and over the age of 18 in the sample population. In China, children under the age of 6 are considered pre-schoolers who are normally enrolled in kindergarten, and the legal age of majority is 18. Therefore, people younger than 6 and older than 18 were excluded from the sample population. It should be noted that, the age reported in this study corresponds to the actual month and year of the testing. Observations with invalid data, such as those with missing age information, were also removed from the sample population. The final sample consisted of 3,955 children aged 6–17 (1,932 boys and 2,023 girls) for fitting the growth and development curves. Our data were collected between August and October of 2019, and the reference time for the seventh national population census was zero hour on November 1, 2020 (16). The ratio of the sampled population to the overall population (including Han ethic population) of Tibet is presented in Table 1 for reference purposes.

**TABLE 1:** Ratio of 2019’s sampled population to 2020’s overall population in Tibet.

Age (years)	Sex	Sampled population	Overall population	Ratio (%)
5-9	M	974	14,203	6.86
	F	1,000	13,856	7.22
10-14	M	909	12,299	7.39
	F	963	11,867	8.11
15-19	M	49	11,794	0.42
	F	60	11,265	0.53

F, female; M, male.

### Measurements

**FIGURE 1:**
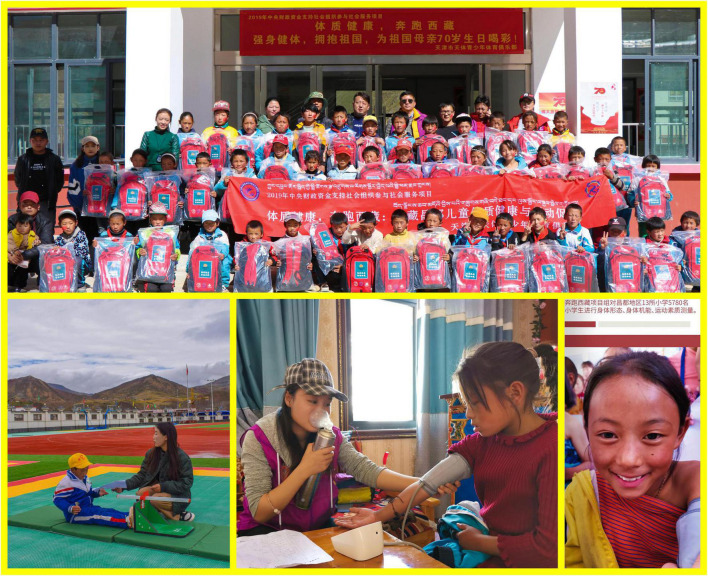
Scenes from the data collection in Jomda County between August and October 2019.

The research team was comprised of more than 50 professors and students from Tianjin University of Sport. Before going for Tibet, all members of the research team were trained and their measuring techniques were standardized against an anthropometry expert and the Chinese National Standards for Student Physical Fitness (17). The Ministry of Education published this national standard in 2014, mandating annual physical fitness testing for all Chinese students from primary school through university in order to monitor their growth and development. Before each testing day, research members calibrated all equipment utilized in this study in accordance with the manufacturer’s instructions. Figure 1 depicts the scenes of data collection at that time.

Included in anthropometric measurements were height, weight, and body mass index. Participants’ height was measured in bare feet to the nearest 0.1 cm, while their weight was measured to the nearest 0.1 kg.

Included in body function assessments were heart rate, blood pressure, and lung function. Prior to measuring heart rate and blood pressure, participants sat still in a quite room for five minutes to allow their heart rate to return to a normal range. The heart rate, systolic blood pressure, and diastolic blood pressure were recorded using a portable electronic blood pressure monitor. The forced vital capacity was used to assess lung function. After fully inhaling, participants were required to exhale until they were exhausted. Each participant was given the assessment three times, with their best performance from the three attempts being recorded.

Included in physical fitness assessments were sit and reach test and standing long jump test. During the sit and reach test, participants were instructed to assume a sitting position with their knees fully extended and their feet firmly placed against a vertical support. Participants reached forward, sliding the hands along the measuring scale three times, with the score corresponding to the longest distance. For the standing long jump test, participants placed their feet together behind a floor-marked starting line and then jumped forward. After three attempts, participants’ longest distance from the starting position to the closest point of landing contact was recorded.

### Modeling

GraphPad Prism version 9.0.0 was utilized for descriptive statistics and the D’Agostino-Pearson normality test. The centile estimation and curve fitting of growth and development were modeled using the *RStudio* version 2022.07.1 + 554 and *gamlss* version 5.4-3.

According to Cole’s original Lambda-Mu-Sigma (LMS) method (10), the first step of centile estimation consists of selecting an appropriate power transformation for age, which can be conveniently determined using the function *lms()* in *gamlss*. The power transformation for age is a necessary step for specific age ranges associated with exponential child growth. From a statistical standpoint, if a power transformation for age is applied, it marginally improves the overall fit of the model in our population. However, such transformation for research purposes could compromise longitudinal analyses of growth and development in this region. For instance, an updated data collection and evaluation in 2025 may require a different power transformation for age than this study, which complicates comparison with this set of data and runs counter to the purpose of this project, which is to establish ready-to-use reference values for practitioners such as educators, clinicians, and health policymakers. For this reason, we did not perform the power transformation for age.

The initial step in a GAMLSS modeling is the selection of a suitable distribution for the response variable, which consists of the measured data on anthropometric indices, body function, and physical fitness. In order to match the sit and reach test that contains negative data, we compared flexible (interval: −∞, ∞) data-fitting distributions skew *t* type 3 (18), skew exponential power type 3 (19), and original Sinh-Arcsinh (20). Box-Cox Cole and Green (12), Box-Cox power exponential (13), and Box-Cox *t* (21) distributions were evaluated to fit the remaining data. After models were fitted with different GAMLSS families of distribution, the best-fitting distribution was chosen using the smallest Schwarz Bayesian criterion (22) value as the measure of disparity.

Next in the GAMLSS modeling is determining the appropriate amount of smoothness for each distribution hyper-parameter. The model was re-fitted using a univariate P-spline smoothing function, and the local maximum likelihood method and the local generalized Akaike information criterion method with penalty κ = 3 (23) were compared to estimate the smoothing degrees of freedom. Again, the suitable model was chosen based on the principle of smallest Schwarz Bayesian criterion value.

Following the selection of the distribution and smoother, the *Q* statistics (24) were used to determine the local goodness of fit for each fitted model. Possible inadequacies in hyper-parameters were resolved by modifying the link functions in the specific distribution or by increasing the degrees of freedom used in the smoothing function. The global goodness of fit of the adjusted models was validated using a visual examination of worm plots (25), statistical validation of normalized quantile residuals, and the Shapiro–Wilk test. As the final step, the centile estimations were calculated.

## Results

The descriptive data of all measurements are summarized in Table 2, which justifies the subsequent modeling with GAMLSS. Following diagnostic procedures, it was determined that the models of height for boys, height for girls, forced vital capacity for girls, and sit and reach test for girls exhibited potential model misfit (Table 3).

**TABLE 2:** Mean ± standard deviation of measurements in Tibet ethnic children aged 6–17.

Age (years)	Sex	Anthropometric indices			Body function				Physical fitness	
		Height (cm)	Weight (kg)	BMI (kg/m^2^)	HR (bpm)	SBP (mmHg)	DBP (mmHg)	FVC (mL)	S&R (cm)	SLJ (cm)
6	M	118 ± 8*	21.6 ± 3.9*	15.4 ± 1.0	89 ± 20	101 ± 19	66 ± 15	967 ± 683*	2 ± 7	96 ± 19*
	F	116 ± 7*	20.8 ± 3.5*	15.4 ± 1.3*	80 ± 21	101 ± 26*	72 ± 18*	750 ± 428*	3 ± 8*	92 ± 17*
7	M	120 ± 6	23.0 ± 3.5*	15.9 ± 1.7*	90 ± 21	104 ± 16*	70 ± 19*	1012 ± 314	4 ± 6*	110 ± 17
	F	120 ± 7	22.2 ± 3.4*	15.2 ± 1.3*	92 ± 17	100 ± 14*	67 ± 15*	898 ± 290*	5 ± 7*	101 ± 14
8	M	124 ± 7*	24.4 ± 4.1*	15.9 ± 1.6*	89 ± 17	103 ± 14*	67 ± 15*	1108 ± 365*	3 ± 7*	117 ± 18
	F	124 ± 7	23.9 ± 4.5*	15.6 ± 1.8*	92 ± 18	100 ± 14*	67 ± 14*	986 ± 385*	5 ± 5*	108 ± 14
9	M	130 ± 8*	27.4 ± 5.7*	16.2 ± 2.0*	92 ± 16*	105 ± 13*	67 ± 13*	1249 ± 467*	4 ± 6*	127 ± 19
	F	130 ± 8*	27.2 ± 5.4*	15.9 ± 1.8*	91 ± 17	103 ± 13*	67 ± 14*	1125 ± 349*	5 ± 5*	115 ± 19*
10	M	134 ± 8*	29.7 ± 5.8*	16.4 ± 1.8*	90 ± 18	107 ± 13*	69 ± 15*	1386 ± 429*	5 ± 5*	137 ± 18*
	F	135 ± 9*	29.1 ± 6.1*	15.9 ± 1.8*	92 ± 18	106 ± 13*	70 ± 15*	1244 ± 434*	6 ± 5*	122 ± 18*
11	M	139 ± 9*	31.9 ± 6.3*	16.5 ± 2.0*	88 ± 17	106 ± 12	66 ± 12*	1586 ± 476*	4 ± 6*	144 ± 20
	F	140 ± 9*	32.5 ± 7.6*	16.5 ± 2.3*	88 ± 15	106 ± 12*	67 ± 12*	1404 ± 450*	6 ± 5*	128 ± 17
12	M	141 ± 10*	33.9 ± 8.1*	16.9 ± 2.3*	83 ± 15	106 ± 12	66 ± 12*	1585 ± 527*	4 ± 6*	148 ± 23*
	F	143 ± 9	34.3 ± 7.1	16.6 ± 2.0*	90 ± 18	108 ± 13*	69 ± 15*	1515 ± 445	7 ± 5*	130 ± 16
13	M	145 ± 10	36.6 ± 7.4*	17.2 ± 2.0*	85 ± 15	109 ± 12	68 ± 15*	1757 ± 546*	5 ± 6	155 ± 23
	F	147 ± 9*	38.0 ± 8.1	17.5 ± 2.4	90 ± 17	109 ± 12	66 ± 10	1552 ± 500	6 ± 6	128 ± 18*
14	M	150 ± 10	38.7 ± 7.5*	17.0 ± 2.0*	85 ± 13	108 ± 11	66 ± 11*	1970 ± 600	3 ± 6	162 ± 23
	F	150 ± 8	41.1 ± 8.6	18.1 ± 2.7*	88 ± 16	107 ± 11	65 ± 9	1698 ± 520*	7 ± 6	130 ± 15
15	M	157 ± 10	44.8 ± 10.1*	18.0 ± 2.4*	84 ± 14	115 ± 16	67 ± 10	2068 ± 689	4 ± 7	174 ± 28
	F	150 ± 10	42.7 ± 9.1	18.6 ± 2.6	83 ± 15	108 ± 10	68 ± 13*	1986 ± 584	8 ± 6	137 ± 14
16	M	155 ± 13	44.3 ± 14.1	18.1 ± 3.8*	89 ± 21	111 ± 14	68 ± 12	1944 ± 754*	5 ± 5	170 ± 28
	F	154 ± 7*	43.1 ± 7.9	18.1 ± 2.5	87 ± 15	110 ± 15	65 ± 10	1758 ± 558	7 ± 6	137 ± 16
17	M	163 ± 11*	48.9 ± 9.0*	18.2 ± 1.1*	80 ± 21*	105 ± 7*	53 ± 7*	1891 ± 300*	-1 ± 8*	203 ± 31*
	F	152 ± 9	45.2 ± 9.8	19.5 ± 2.4*	90 ± 13	112 ± 11	66 ± 10	1951 ± 718	7 ± 4	137 ± 18*

BMI, body mass index; DBP, diastolic blood pressure; F, female; FVC, forced vital capacity; HR, heart rate; M, male; SBP, systolic blood pressure; SLJ, standing long jump test; S&R, sit and reach test.

*Denotes data for age are non-Gaussian.

**TABLE 3:** Summary of the *P* values for the *Q* statistics.

Object	Sex	*Q*_1_	*Q*_2_	*Q*_3_	*Q*_4_
Height	M	0.320	0.754	0.363	0.000
	F	0.226	0.132	0.200	0.807
Weight	M	0.548	0.291	0.919	0.155
	F	0.262	0.310	0.079	0.555
Body mass index	M	0.705	0.271	0.443	0.953
	F	0.290	0.290	0.592	0.418
Heart rate	M	0.504	0.203	0.469	0.707
	F	0.321	0.402	0.737	0.983
Systolic blood pressure	M	0.357	0.830	0.524	0.983
	F	0.557	0.973	0.752	0.933
Diastolic blood pressure	M	0.679	0.530	0.694	0.729
	F	0.077	0.497	0.704	0.956
Forced vital capacity	M	0.345	0.211	0.398	0.622
	F	0.751	0.093	0.014	0.333
Sit and reach test	M	0.144	0.366	0.845	0.066
	F	0.087	0.183	0.393	0.038
Standing long jump test	M	0.261	0.621	0.078	0.109
	F	0.361	0.255	0.608	0.023

F, female; M, male.

**FIGURE 2:**
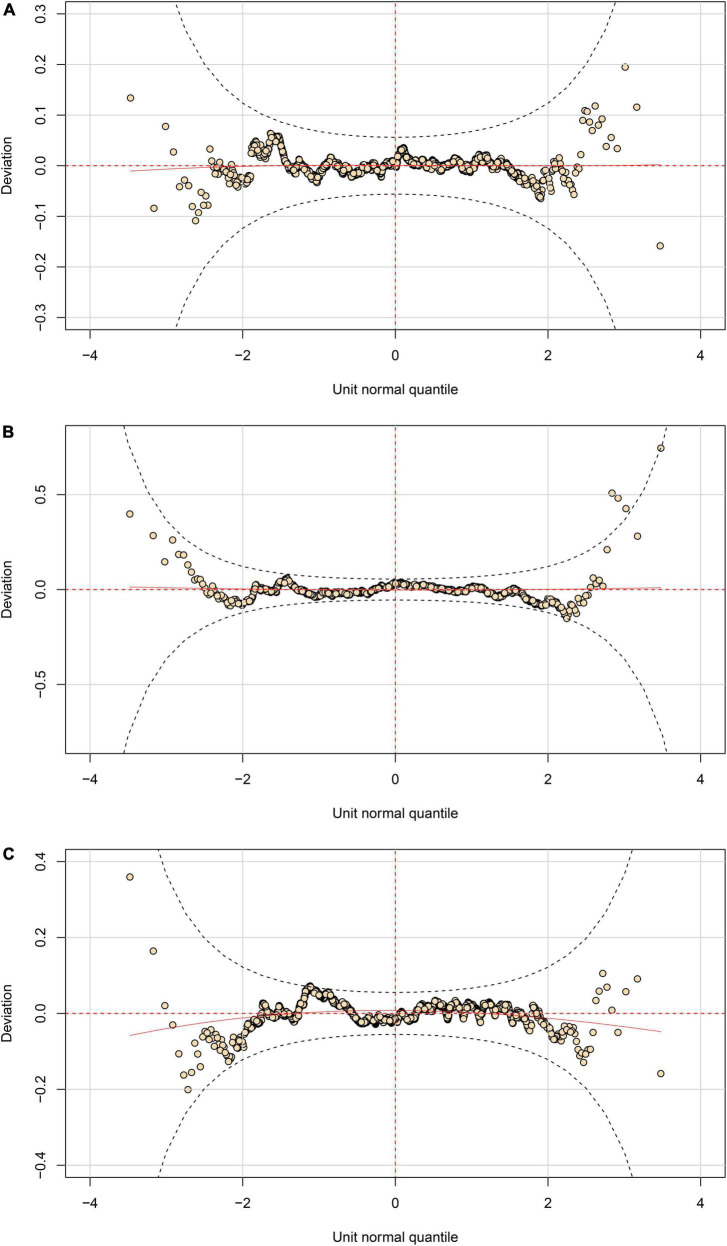
Worm plots of fitted GAMLSS models. **(A)** Height for boys; **(B)** forced vital capacity for girls; **(C)** sit and reach test for girls.

First, the kurtosis of the residuals for models of height for boys and the sit and reach test for girls deviates significantly from a normal distribution. The *P* values of Shapiro–Wilk tests for models of height for boys and the sit and reach test for girls are 0.9558 and 0.3118, respectively, and their shape of worm plots (Figures 2A,C) show that the misfits are situated at the distribution’s tails. These diagnostic tools demonstrate that only the kurtosis is responsible for the local misfit in the *Q* statistics. Cole has argued that kurtosis-controlling in the Box-Cox power exponential and Box-Cox *t* influences the distribution only beyond the 1st and 99th centiles, and that there is little point in modeling kurtosis even when it exists (26). Given that the global goodness of fit is acceptable in this instance, we did not adjust the models of height for boys and the sit and reach test for girls further.

Second, for the model of height for girls, it was the variance of the residuals that deviated significantly from a normal distribution. Accordingly, we adjusted the penalty κ to 3.84 and re-fitted the model, which improved the fit, as confirmed by the adjusted *Q* statistics (*P* value for *Q*_2_ statistics = 0.132 as shown in Table 2) and the Shapiro–Wilk test (*P* = 0.8494).

Third, for the model of forced vital capacity for girls, it is the skewness of the residuals that deviated significantly from a normal distribution. Initially, we attempted to adjust the model by changing the link function from identity to log in the shape hyper-parameter nu, which is related to the skewness of the distribution. This only marginally improved the local goodness of fit (*P* value for *Q*_3_ statistics = 0.014 as shown in Table 2), but it still penalized the fit. We also attempted to increase the degree of freedom for nu, which complicated the model but did not improve its fit. As shown in Figure 2B, some points lie outside the approximate 95% pointwise elliptical confidence bands, indicating that fitted distribution is too skewed to the right. Overall, compensating for skewness is challenging since the smoothing curve cannot adequately capture the age range’s natural oscillations. Given that the Shapiro–Wilk test (*P* = 0.0633) is relatively acceptable, we decide to accept the existing model despite the fact that the hyper-parameter nu did not fit across ages well.

**FIGURE 3:**
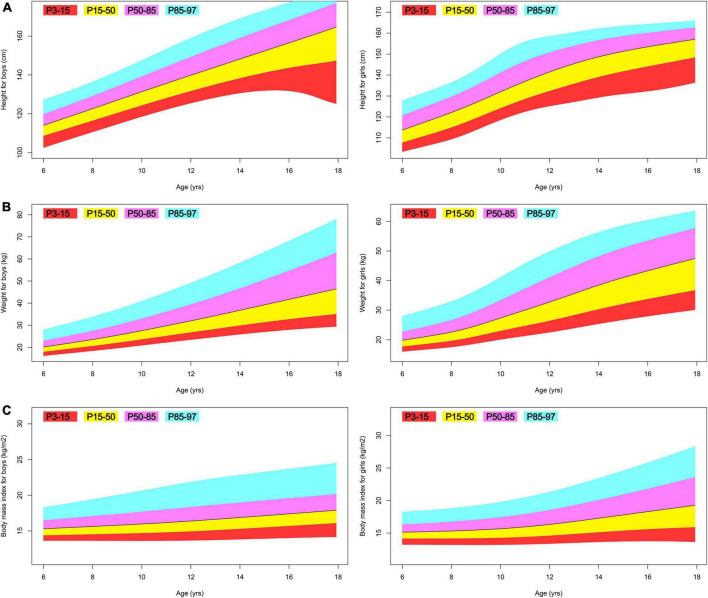
Fan plot displaying the 3, 15, 50, 85, and 97% centiles for the anthropometric indices of Tibetan ethnic children aged 6–17. **(A)** Height; **(B)** weight; **(C)** body mass index.

**FIGURE 4:**
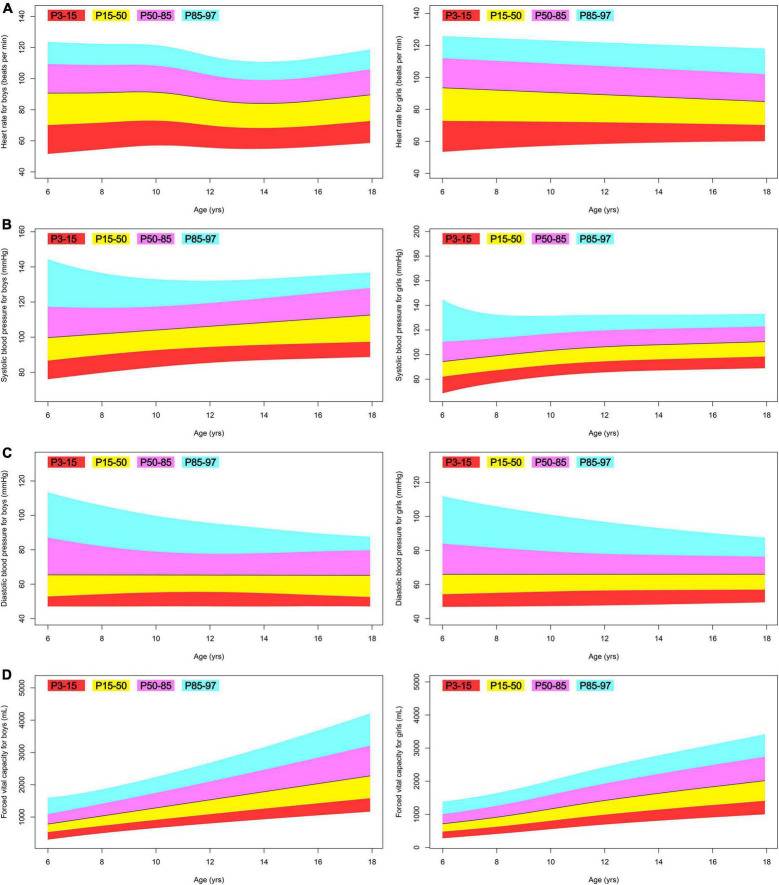
Fan plot displaying the 3, 15, 50, 85, and 97% centiles for the body function of Tibetan ethnic children aged 6–17. **(A)** Heart rate; **(B)** systolic blood pressure; **(C)** diastolic blood pressure; **(D)** forced vital capacity.

**FIGURE 5:**
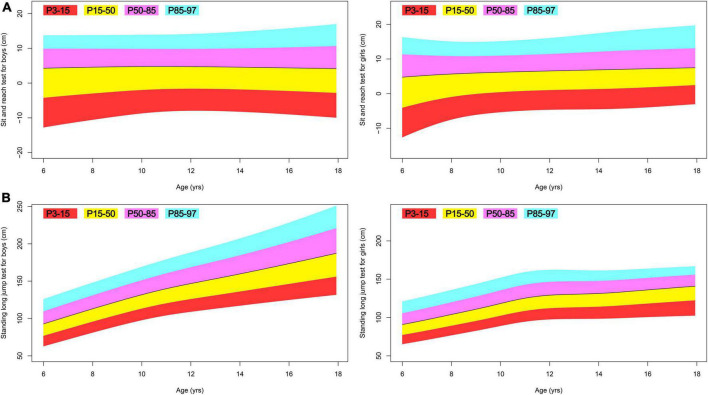
Fan plot displaying the 3, 15, 50, 85, and 97% centiles for the physical fitness of Tibetan ethnic children aged 6–17. **(A)** Sit and reach test; **(B)** standing long jump test.

Table 4 provides a summary of all fitted GAMLSS models. Overall, their normalized quantile residuals have a mean nearly zero, a variance nearly one, a moment-based skewness nearly zero, and a moment-based kurtosis nearly three. Figures 3–5 illustrate centile estimations for the measurements against ages ranging from 6 to 17 years. An extensive presentations comprising tabulated centiles are available on figshare (doi: 10.6084/m9.figshare.20898196.v1).

**TABLE 4:** Summary of fitted GAMLSS model parameters.

Object	Sex	*n*	Distribution	*Pb()* method	*edf*				SBC
					μ	σ	ν	τ	
Height	M	1,919	BCT	ML	2.68	2.00	3.90	2.00	13,328.80
	F	2,015	BCPE	GAIC	4.69	4.35	5.21	2.00	14,183.48
Weight	M	1,918	BCPE	ML	3.84	2.00	2.00	2.00	11,488.57
	F	2,015	BCPE	ML	5.45	4.07	2.00	2.00	12,342.00
Body mass index	M	1,916	BCT	GAIC	2.79	2.93	2.00	2.00	7,275.196
	F	2,012	BCT	GAIC	3.70	2.21	2.00	2.00	7,822.246
Heart rate	M	1,422	BCCG	ML	5.14	2.66	2.00	N/A	12,084.82
	F	1,496	BCCG	GAIC	2.00	2.00	2.00	N/A	12,801.29
Systolic blood pressure	M	1,379	BCPE	GAIC	2.00	3.05	2.00	2.69	10,967.22
	F	1,450	BCT	GAIC	3.23	2.13	2.00	2.00	11,519.72
Diastolic blood pressure	M	1,379	BCPE	ML	2.00	2.91	2.00	4.02	10,860.57
	F	1,449	BCPE	GAIC	2.00	2.00	2.00	2.80	11,354.36
Forced vital capacity	M	1,903	BCT	ML	2.00	2.00	2.00	2.00	28,338.91
	F	1,986	BCT*	ML	4.26	2.00	2.00	2.00	29,289.65
Sit and reach test	M	1,889	SHASHo	GAIC	2.00	2.99	2.53	2.00	12,124.81
	F	1,977	SHASHo	GAIC	2.00	4.01	2.24	2.00	12,398.50
Standing long jump test	M	1,916	BCT	GAIC	3.88	3.31	2.00	2.00	16,830.35
	F	2,004	BCT	GAIC	5.84	2.00	2.81	2.00	16,914.78

BCCG, Box-Cox Cole and Green; BCPE, Box-Cox power exponential; *edf*, effective degrees of freedom; F, female; GAIC, generalized Akaike information criterion; M, male; ML, maximum likelihood; *n*, the number of observations in the fit; *pb()*, P-splines smoothing function in *gamlss*; SBC, Schwarz Bayesian criterion; SHASHo, original Sinh-Arcsinh; μ, mu; σ, sigma; ν, nu; τ, tau.

*The default link function for the shape hyper-parameter nu has been changed from identity to log.

## Discussion

Based on the GAMLSS method, this is the first study in China to provide a comprehensive assessment of the growth and development of Tibetan school-aged children of ethnicity. Traditionally, longitudinal and cross-regional research on anthropometric measurements rely on population means to compare trends and differences. The validity of this approach is questionable. As demonstrated by this research, there are numerous non-Gaussian data for which the mean cannot accurately represent the population state. GAMLSS presupposes that the response variable may be fitted by a general distribution family, giving a versatile and valid method for converting highly skew and/or kurtotic data to normally distributed z-scores. This advanced statistical method enables us to interpret our non-Gaussian data for meaningful comparison with prior data and to develop sensible reference values for a variety of practical diagnosis.

While it is reassuring to observe that nearly all of the children in this study fall within the normal body mass index range (27), we discovered that the height of Tibetan ethnic boys and girls is much shorter than that of Han and other Chinese ethnic minority children. In 2015, Zhang and colleagues collected data from 44,437 students aged 6–17 in Jiamusi and developed LMS-based reference values (28). Jiamusi is situated at an average altitude of 194 m and is home to 41 distinct ethnic groups. Compared to the matching 50% percentile Jiamusi 2015 study, our Tibetan ethnic boys at the 50th percentile are shorter by 10, 11, 12, 13, 14, 16, 20, 22, 23, 21, 18, and 14 cm between the ages of 6 and 17; and, our Tibetan ethnic girls at the 50th percentile are shorter by 9, 10, 11, 12, 14, 16, 17, 15, 13, 11, 9, and 8 cm between the ages of 6 and 17. In 2018, Shi and colleagues collected data from 25475 urban and rural students aged 7–18 in 21 cities and counties in Sichuan and developed LMS-based reference values (29). Chengdu, the largest city in Sichuan, is located at an average altitude of 771 m, while Sichuan’s average altitude is 2,408 m. The majority of Sichuan’s population is of Han ethnicity. Compared to the matching 50th percentile Sichuan 2018 study (rural students), our Tibetan ethnic boys at the 50th percentile are shorter by 6, 5, 6, 7, 8, 10, 13, 14, 14, 12, and 9 cm between the ages of 7 and 17; and, our Tibetan ethnic girls at the 50th percentile are shorter by 4, 6, 7, 8, 9, 9, 9, 8, 6, 5, and 3 cm between the ages of 7 and 17. Based on these cross-regional comparisons, it is unequivocal that high altitude above 3,000 m has a deleterious effect on the early childhood height growth (3) of Tibetan ethnic children that persists till the age of 17 in this study.

Human growth is affected by genetic, environmental, dietary, and hormonal factors, and stunted growth in Tibetan children may have multiple causes, some of which may be ameliorated by nutritional interventions. Vitamin D regulates calcium and phosphorus metabolism to facilitate bone growth and mineralization, and data show that vitamin D insufficiency is associated with pediatric stunting (30). Recent research indicates that the serum 25-hydroxy vitamin D level in Tibetan children under the age of 12 was only 19.7 ng/ml (31), and this subclinical deficiency is likely a cause of childhood stunting in Tibet. Dermience et al. (32) have evaluated the mineral and trace element consumption of pre-school children living in rural areas of Tibet. It was revealed that Tibetan children aged 3–5 did not achieve the recommended dietary intakes for the majority of the examined elements, and that their K, Ca, Zn, Cu, and Se intakes were inadequate, which could affect bone metabolism and their growth in height. Since the early 2000s, the nutrition status of Tibetan children has been a known issue (33), and the Chinese government has increased fiscal support and implemented targeted nutrition grants in an effort to reduce malnutrition among Tibetan children (34). The effect of these measures will be investigated in future longitudinal studies.

A subset of children in this study had low blood pressure of non-clinical importance, which was also identified previously (35). Comparing the 50th percentile blood pressure in this study to the matching data from a LMS-based reference data (36), which included 5,200 multi-ethnic Chinese children residing at Ürümqi (altitude: 800 m) and Altay (altitude: 1,442 m) in 2005, we find no difference between the two sample populations. Overall, the Tibetan children in this study demonstrate normal heart rate and blood pressure regulation. While there is a clear association between increasing altitude and the prevalence of hypertension among Tibetans (37), we argue that there are other key factors, likely unhealthy diets with excessive salt intake (32), which do not manifest in children but gradually drive the occurrence of hypertension in adults (38).

This study presents the first systematic comparison of lung function between high-altitude residents and other Chinese children, and the results are striking. Zhu et al. (39) reported the LMS-based forced vital capacity reference values, which are based on the same sample population (28) as was discussed previously. Compared to the matching 50th percentile Jiamusi 2015 study, our Tibetan ethnic boys at the 50th percentile are lower by 39, 147, 312, 568, 889, 1,221, 1,462, 1,594, and 1,668 ml between the ages of 9 and 17; and, our Tibetan ethnic girls at the 50th percentile are lower by 23, 77, 138, 220, 329, 456, 548, 589, and 602 ml between the ages of 9 and 17. In 2017, Li and colleagues collected data from 63,805 students aged 6–18 in Zibo and developed LMS-based reference values (40). Zibo is located at an average altitude of 67 m, and the majority of its population is Han. Compared to the matching 50th percentile Zibo 2017 study, our Tibetan ethnic boys at the 50th percentile are lower by 189, 284, 395, 521, 683, 902, 1,172, 1,467, 1,724, 1,855, 1,887, and 1,882 mL between the ages of 6 and 17; and, our Tibetan ethnic girls at the 50th percentile are lower by 157, 240, 326, 435, 580, 737, 867, 930, 922, 854, 767, and 691 ml between the ages of 6 and 17. Notably, none of the children in this study had lung illnesses.

At high altitude, lung function is accompanied by a decrease in gas exchange and oxygen diffusion from the air into the blood (41), and Tibetans are believed to have acquired adaptive lung functions (42). The mean sea-level air density is 1,225 g/m^3^ and the standard atmospheric pressure is 1,013.2 hPa, while the air density in Jomda County is 810 g/m^3^ and the standard atmospheric pressure is 652 hPa, which corresponds to 66 and 64% of the density and pressure at sea level, respectively. Even without adjusting for height and weight, the aforementioned comparisons of lung function with low-altitude, multi-ethnic Chinese population suggest a worrisome condition among Tibetan ethnic children in the investigated area. Based on our first-hand field observations, the potential cause may be a lack of structured exercise programs and qualified physical educators. The studied population resides at an average altitude of 3,650 m, and the hypoxic environment and harsh climate limit the amount of time and types of outdoor sports that students can participate in. Furthermore, the vast majority of physical educators in local schools are part-time teachers of other curriculum who lack formal physical education training. Inadequate physical education for Tibetan children may impede their physical fitness development, especially childhood lung function, in light of these severe constraints.

This growth and development deficiency is recommended to be addressed by two solutions. It has been found that a decrease in respiratory muscle strength may contribute to the decrease in the forced vital capacity reported at high altitude (43). Naturally, the short-term solution may involve enhancing respiratory muscle exercise. Azab et al. (44) reported recently that concurrent chest and chest expansion exercises enhanced respiratory muscle strength and lung function in children with congenital diaphragmatic hernia. Although such a training effect has never been studied in healthy children residing at altitudes, it is worthwhile to at least include upper body resistance training, which is generally beneficial at enhancing forced vital capacity (45). Obviously, this concept cannot be effectively implemented without physical education and sports training professionals. Therefore, Tibetan regions require increased government investment in their educational resources. We recommend that the Ministry of Education and General Administration of Sport create a special grant to entice outstanding young physical educators to work in Tibet for a set period of time (e.g., one year), and that the fiscal impact could be multiplied by using sports as an additional means to improve the health of Tibetan children.

There are numerous published reports on the physical fitness of multi-ethnic, school-aged Chinese children residing at low altitudes. As previously stated, we caution against direct comparisons based on means, which can be misleading if the distribution of the data is unknown. Evident from this study is the value of data analysis with GAMLSS for the quick and accurate identification of growth insufficiency, which has important clinical significance. Using our reference values, for instance, a doctor may use the 15th percentile as a diagnostic criterion for short stature at a particular age and give prompt intervention methods. The same principle applies to the physical fitness tests, which are currently graded according to the Chinese National Standards for Student Physical Fitness (17). However, this national standard poses problems. The 10-to-100 score scale shows a pattern of increasing scores, and based on our expertise in the field of sports statistics, it is quite unlikely to observe a physiological shift in a pattern of variance that is so stable. The national standard was last revised in 2014, and we believe that during the standard’s development, the expert committee did not have access to a database as comprehensive as the one available now, which covers big data. Therefore, we recommend that the Ministry of Education and General Administration of Sport adopt the GAMLSS method for the future revision of the national standard for physical fitness, which will significantly enhance the scientific and practical aspects of the standard.

Cole suggests that, for an appropriate centile study, the sample size for a typical study spanning 0–20 years of age should include at least 2,000 observations (26). In this study, the centile estimations were developed using data from 1,932 boys and 2,023 girls aged 6–17. In addition, this set of reference values is supported by the fact that our population between the ages of 6 and 14 comprises a sizable proportion of all Tibet’s children. This collection of reference values has major statistical implications for 6 to 14-year-old Tibetan children residing at an altitude of 3,650 m. In contrast, for children aged 15–17, it represents less than 0.5% of the total Tibetan children in this age range. The statistical under-power can be clearly reflected from the weight for boys (see also Figure 3A trajectory), which is locally supported by fewer and more outlier data and exhibits a biologically abnormal fall in this age range. Therefore practitioners should exercise caution when considering the practical inferences for this age range.

In conclusion, this study established the first scientific reference values for assessing the growth and development of 6- to 17-year-old Tibetan ethnic children living at an altitude of 3,650 m. Our reference values can assist practitioners with accurate diagnosis and early intervention. Researchers can utilize these reference values for longitudinal studies to monitor population health. We provide Chinese policymakers with intervention plans for resolving some of the concerns found in the data and a framework for revising the national standard to be more scientific. We envision the impact of this work will be broad and profound in the next years.

## Data availability statement

The datasets that support the findings of this study are available from the corresponding author upon reasonable request.

## Ethics statement

This research was approved by the Tianjin University of Sport. Written informed consent was obtained from the individuals, and minors’ legal guardian/next of kin, for the publication of any potentially identifiable images or data included in this article.

## Author contributions

All authors listed have made a substantial, direct, and intellectual contribution to the work, and approved it for publication.
